# High CRP values predict poor survival in patients with penile cancer

**DOI:** 10.1186/1471-2407-13-223

**Published:** 2013-05-03

**Authors:** Sandra Steffens, Andreas Al Ghazal, Julie Steinestel, Rieke Lehmann, Gerd Wegener, Thomas J Schnoeller, Marcus V Cronauer, Florian Jentzmik, Mark Schrader, Markus A Kuczyk, Andres J Schrader

**Affiliations:** 1 Department of Urology, Hannover Medical School, Hannover, Germany; 2 Department of Urology, Ulm University Medical Center, Pritzwitzstrasse 43, Ulm, D-89075, Germany; 3 Cancer Center, Hannover Medical School, Hannover, Germany

**Keywords:** SCC, Penis, Penile cancer, Biomarker, C-reactive protein, Prognosis, Survival

## Abstract

**Background:**

High levels of circulating C-reactive protein (CRP) have recently been linked to poor clinical outcome in various malignancies. The aim of this study was to evaluate the prognostic significance of the preoperative serum CRP level in patients with squamous cell carcinoma (SCC) of the penis.

**Methods:**

This retrospective analysis included 79 penile cancer patients with information about their serum CRP value prior to surgery who underwent either radical or partial penectomy at two German high-volume centers (Ulm University Medical Center and Hannover Medical School) between 1990 and 2010. They had a median (mean) follow-up of 23 (32) months.

**Results:**

A significantly elevated CRP level (>15 vs. ≤ 15 mg/l) was found more often in patients with an advanced tumor stage (≥pT2) (38.9 vs. 11.6%, p=0.007) and in those with nodal disease at diagnosis (50.0 vs. 14.6%, p=0.007). However, high CRP levels were not associated with tumor differentiation (p=0.53). The Kaplan-Meier 5-year cancer-specific survival (CSS) rate was 38.9% for patients with preoperative CRP levels above 15 mg/l and 84.3% for those with lower levels (p=0.001). Applying multivariate analysis and focusing on the subgroup of patients without metastasis at the time of penile surgery, both advanced local tumor stage (≥pT2; HR 8.8, p=0.041) and an elevated CRP value (>15 mg/l; HR 3.3, p=0.043) were identified as independent predictors of poor clinical outcome in patients with penile cancer.

**Conclusions:**

A high preoperative serum CRP level was associated with poor survival in patients with penile cancer. If larger patient populations confirm its prognostic value, its routine use could enable better risk stratification and risk-adjusted follow-up of patients with SCC of the penis.

## Background

Squamous cell carcinoma (SCC) of the penis accounts for more than 95% of penile cancer cases. Though relatively rare in the Western world, its incidence has increased slightly with important variations in several European regions, ranging from 0.5 to 1.6 per 100,000 men annually. Penile cancer has a much higher incidence rate in the non-Western world (e.g. Uganda or Brazil), where it comprises up to 10% of all malignant diseases in men [1,2].

Several prognostic factors have been established for patients with penile cancer. Nodal metastasis is undoubtedly the most important predictor of poor clinical outcome. Additional factors implicated in impaired survival include advanced local tumor stage, perineural and lymphovascular invasion, anatomic site, size, growth pattern, and high histologic grade [2]. Classical molecular markers are not clinically useful in SCC of the penis. SCC antigen lacks the sensitivity needed to detect a small tumor burden and has little prognostic significance for survival after surgery [3]. A poor prognosis and the detection of lymph node metastases has been associated with the overexpression of p53 and Ki-67, as well as loss of membraneous E-cadherin, but these markers are not useful in clinical practice [2,4].

C-reactive protein (CRP) is an acute-phase reactant produced almost exclusively by the liver. Plasma CRP levels can increase as much as 1000-fold in response to microbial infection, trauma, infarction, autoimmune diseases or malignancies. Elevated CRP levels may be due to underlying malignancy or premalignancy or to tissue inflammation associated with tumor growth [5]. However, it is still unclear whether the tumor promotes inflammation or is rendered more aggressive by it. High levels of circulating CRP have recently been linked to poor prognosis in various malignancies, including oral SCC [6], esophageal SCC [7,8], non-small cell lung cancer [9], small cell lung cancer [10], melanoma [11], hepatocellular carcinoma [12,13], breast cancer [14], endometrial cancer [15], renal cell carcinoma [16,17], urothelial carcinoma [18], castration-resistant prostate cancer [19], and even diffuse large B cell lymphoma [20]. There have been conflicting reports about the correlation between CRP and prognosis in patients with colorectal cancer [21,22].

The aim of this retrospective two-center study was to evaluate the impact of CRP levels at diagnoses on the prognosis of penile cancer patients.

## Methods

### Patient and tumor characteristics

This study included 79 patients with information about their CRP value directly prior to (partial) penectomy who underwent penile cancer surgery from 1990 to 2010 at the Ulm (n=43) or Hannover (n=36) University Medical Centers. The study was approved by the Ulm University ethics committee (proposal no. 241/12). All research has been carried out according to the current Helsinki Declaration (59^th^ edition, Seoul, Korea, 2008; http://www.wma.net/en/30publications/10policies/b3). The histological tumor subtype was determined according to the 2010 UICC Classification. Our institutional databases were used to obtain patient and tumor characteristics, such as age, stage, regional lymph node involvement or distant metastasis, histological subtype, tumor grade, CRP value, and body mass index (BMI).

### Follow-up

The length of follow-up was calculated as the time from surgery to the time of death or last follow-up. Death was assessed as either cancer-related or non-cancer-related. The primary end point of this study was cancer-specific survival (CSS). Information about the exact date and cause of death was obtained in each case from the general practitioner, from a close family member or from the hospital records of patients who had been followed up or had died in one of our institutions. Follow-up assessment ended in July 2012. Until that time, all patient data were regularly updated at least every 6 months.

### Statistical methods

Continuous variables were reported as the mean and the standard deviation (SD) for parametric distribution or as the median and the interquartile range (IQR) for non-parametric distribution. Chi-square and Fisher’s exact tests were conducted to assess correlations between covariate distributions and nodal disease. Mann-Whitney tests were applied to compare continuous cardinal parameters between the two groups. The optimum CRP cut off value to predict prognosis was calculated using receiver operating characteristics (ROC) analysis referring to cancer specific death.

Kaplan-Meier analysis was performed to estimate survival time, and subgroups were compared using the log rank test. Multivariate Cox regression models were used to assess the association between survival and CRP levels adjusted for different clinical and patient covariates (age, tumor stage and grade, and the metastatic status). SPSS 19.0 was used for statistical analysis. A two-tailed p value less than 0.05 was considered significant in all tests.

## Results

### Patient and tumor characteristics

Our patient population of 79 men, aged 33-92, median (mean) 65.2 (65.4) years, presented with SCC of the penis and had penile cancer surgery. Sixty-four of them also underwent inguinal lymphadenectomy.

During the median (mean) follow-up of 23.0 (31.9) months, 14 patients died of penile SCC, and 8 succumbed to other causes.

The median body mass index (BMI) for all patients was 26.6 kg/m^2^ (IQR, 23.8 – 29.0), and the median (mean) preoperative CRP value of all evaluable patients (n=79) was 4.0 (15.0) mg/l. Preoperative CRP values were normal (<5 mg/l) in 45 patients (57.0%) and elevated in 34 (43.0%). At the time of penile surgery, there were 36 patients (45.6%) with locally advanced penile cancer (≥pT2), 16 (25.0%) with nodal involvement, and 4 (5.4%) with distant metastasis. All patients with visceral/distant metastasis also presented with nodal involvement.

Table 1 gives a detailed summary of patient and tumor characteristics, including stage and grade.

**Table 1:** Association between different patient and cancer-specific variables with CRP elevation prior to penile cancer surgery

**Variable**	**CRP ≤ 15 mg/l**	**CRP > 15 mg/l**	**p-value**
Age, median [years] (IQR)^1^	64.8 (58-71)	70.3 (62-77)	0.07
BMI, median [kg/m^2^] (IQR)^1^	26.6 (24.1-29.4)	26.1 (21.9-28.3)	0.19
Stage			0.012
pTis	11 (18.3%)	1 (5.3%)	
pTa	3 (5.0%)	1 (5.3%)	
pT1	24 (40.0%)	3 (15.8%)	
pT2	17 (28.3%)	6 (31.6%)	
pT3	4 (6.7%)	5 (26.3%)	
pT4	1 (1.7%)	3 (15.8%)	
Nodal metastasis^1^			0.007
N0	41 (83.7%)	7 (46.7%)	
N+	8 (16.3%)	8 (53.3%)	
Grade			0.53
G1	11 (22.9%)	2 (11.1%)	
G2	25 (52.1%)	10 (55.6%)	
G3/4	12 (25.0%)	6 (33.3%)	

^1^ at time of penile cancer surgery. Abbreviations: CRP = C-reactive protein, N = nodal status.

### Clinical outcome

**Figure 1:**
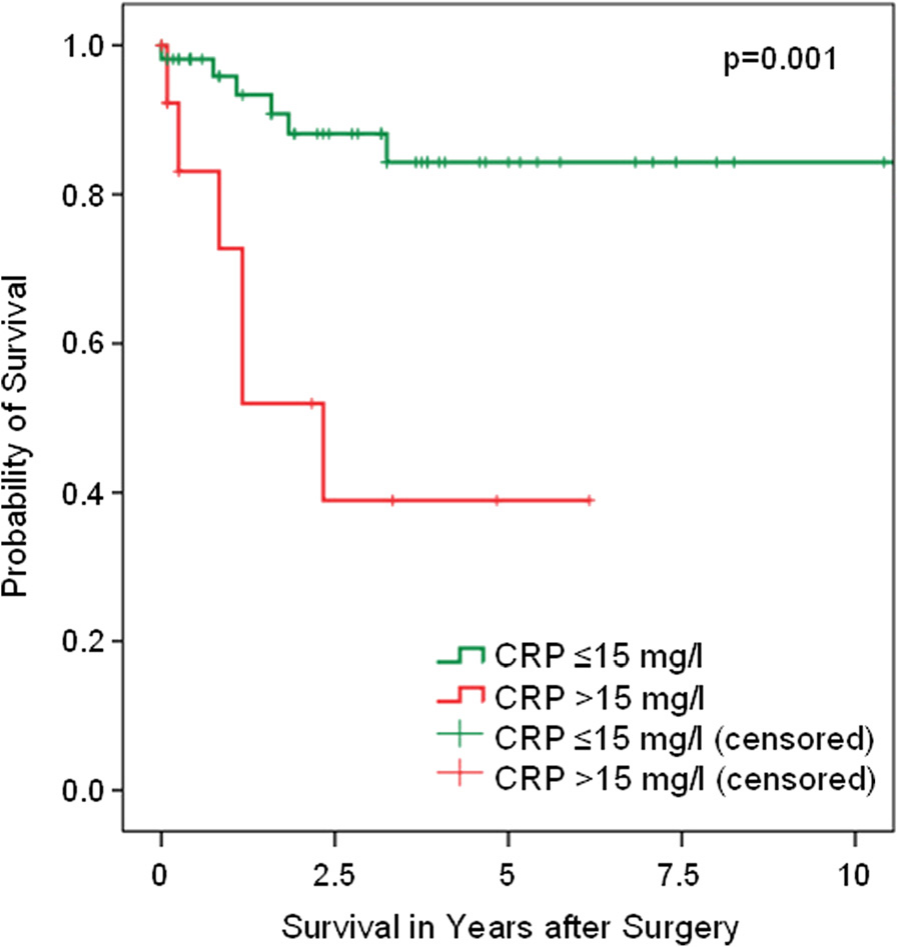
**Cancer-specific survival (Kaplan-Meier) of patients with penile SCC plotted against the preoperative CRP group.** The 5-year survival rate of all evaluable patients (n=69) was 84.3% for CRP ≤15 mg/l (n=54) and 38.9% for CRP >15 mg/l (n=15) (p=0.001, log rank).

Univariate Cox regression analysis showed that – in contrast to older age (>median; HR 0.52, 95% CI 0.16-1.73, p=0.30), elevated BMI (>25 kg/m^2^; HR 0.51, 95% CI 0.16-1.77, p=0.29), and even high tumor grade (≥G3; HR 1.63, 95% CI 0.48-5.49, p=0.43) - both high tumor stage (≥pT2; HR 17.16, 95% CI 2.21-133.34, p=0.007) and metastasis at diagnosis (HR 12.02, 95% CI 3.23-44.79, p<0.001) were associated with poor CSS. An elevated CRP value also proved to be a prognosticator of poor CSS, regardless of the cut-off level. However, using ROC analysis a CRP cut-off of 15 mg/l was found to be optimal for achieving high prognostic accuracy. Accordingly, with a hazard ratio (HR) of 5.58 (95% CI 1.79-17.42, p=0.003), the CRP cut-off of 15 mg/l was superior to alternative cut-offs of 5 mg/l (HR 3.55, 95% CI 1.07-11.85, p=0.039), 10 mg/l (HR 4.59, 95% CI 1.47-14.31, p=0.009), or 20 mg/l (HR 4.18, 95% CI 1.32-13.22, p=0.015). The 5-year survival rate of all evaluable patients (n=69) was 84.3% for CRP ≤15 mg/l (n=54) and 38.9% for CRP >15 mg/l (n=15; p=0.001, log rank; Figure 1).

### Associations between patient or tumor characteristics and the CRP level

The median (mean) CRP value was 3.9 (4.1) in the subgroup with CRP ≤15 and 41.0 (49.3) mg/l in one with CRP >15 mg/l. The two groups had a comparable median BMI (Table 1), but patients with a higher CRP level tended to be slightly older (median, 70.3 vs. 64.8 years; p=0.069; Mann-Whitney-U test). Moreover, the CRP level correlated significantly with the tumor stage: 73.7% of all patients with CRP >15 and 36.7% of those with CRP ≤15 mg/l suffered from locally advanced (pT≥2) penile cancer (p<0.007, Fisher’s exact test). The risk of presenting nodal disease (53.3% vs. 16.3%, p=0.007) or distant metastasis (11.8% vs. 3.5%, p=0.22) was also higher in the CRP >15 mg/l group. However, the presurgical CRP level did not correlate with tumor differentiation: 33.3% of all patients with CRP >15 and 25.0% of those with CRP ≤15 mg/l presented with poorly differentiated (≥G3) cancer (p=0.53).

### Independent predictors of cancer-specific survival

Multivariate regression analysis showed that - unlike age and tumor grade - nodal metastasis at the time of surgery was a significant and independent predictor of poor CSS in patients with penile cancer. In contrast, a tumor stage ≥pT2 and a CRP value >15 mg/l failed to reach statistical significance. These results did not change when applying step-wise backward LR-regression analyses. Here too, only metastasis at the time of penile surgery remained as an independent predictor of cancer specific survival (HR 7.65, 95% CI 2.04-28.7, p=0.003, Cox regression analysis). However, focusing on the subgroup of patients without metastasis, both advanced local tumor stage (≥pT2; HR 8.78, 95% CI 1.1-70.7, p=0.041) and an elevated CRP value (>15 mg/l; HR 3.34, 95% CI 1.04-10.7, p=0.043) were identified as predictors of poor clinical outcome in patients with penile cancer (Table 2).

**Table 2:** Focusing on penile cancer patients without metastasis at the time of penile surgery multivariable analysis revealed that both tumor stage and the CRP level were independent prognostic markers for cancer-specific survival

**Variable**	**HR (95% CI)**	**p-value**
Age [years]^2^	0.99 (0.94-1.05)	0.82
Stage		0.04
pT<2	1 (Reference)	
pT≥2	8.78 (1.09-70.66)	
Grade		0.82
G1/2	1 (Reference)	
G3/4	1.15 (0.33-3.99)	
CRP-value^1^		0.04
CRP≤ 15 mg/l	1 (Reference)	
CRP> 15 mg/l	3.34 (1.04-10.72)	

^1^ at time of penile cancer surgery. Abbreviations: HR = hazard ratio, CRP = C-reactive protein.

## Discussion

C-reactive protein (CRP) is an acute-phase reactant that is elevated during bacterial infection, inflammatory disease, trauma, surgery, and cancer. It is mainly produced by the liver in response to an inflammatory stimulus involving increased cytokine expression [23]. Elevated plasma CRP levels are also associated with an increased risk of cancer [24-27], but causality has not been established. High levels of circulating CRP have also been linked to advanced disease and a poor prognosis in various malignancies, including oral and esophageal SCC [6-8], lung cancer [9,10], melanoma [11], hepatocellular carcinoma [12,13], breast cancer [14], endometrial cancer [15], renal cell carcinoma [16,17], urothelial carcinoma [18], and castration-resistant prostate cancer [19,28]. This study shows for the first time that elevated preoperative CRP levels are also associated with penile cancer stage, but not grade. Moreover, elevated CRP values were found to indicate poor survival. In multivariate analysis, however, high serum CRP failed to retain significance as an independent prognostic factor for SCC of the penis. This may be due to either the small sample size or the strong association between CRP and metastasis; nodal disease was the only significant predictor of cancer-related death in both uni- and multivariate analyses. In the subgroup of patients without metastasis at the time of penile surgery, however, both advanced tumor stage and an elevated CRP value were identified as independent predictors of poor cancer specific survival.

Taken together, there is a strong association between circulating CRP levels and cancer risk and/or progression, which may be due to (1) causality: elevated CRP levels cause or promote cancer, (2) reverse causality: (occult) cancer increases CRP levels, or (3) confounding: a third factor, e.g. inflammation, increases both CRP levels and the risk of cancer (progression) [29]. The latter theory is now generally accepted for many malignancies, including penile cancer [30]. The second theory has also been supported by several authors who recently demonstrated that tumor cells can express IL-6 and even CRP. Using immunohistochemical analysis, Johnson et al. [31] only recently evaluated the influence of intratumoral CRP on overall survival in 95 patients with localized clear cell RCC. Mean overall survival was significantly longer in the groups with a low (44.2 months) and intermediate (40.5 months) intratumoral CRP staining intensity than in the group with tumors expressing high amounts of CRP (31.6 months; p=0.002 and p=0.067). Applying multivariate analysis, Johnson et al. [31] demonstrated a 12 times higher overall mortality risk for RCC patients with high than for those with low levels of intratumoral CRP. Using immunohistochemistry, Nakatsu et al. [8] detected CRP-expressing tumor cells in 59% of patients with thoracic esophageal SCC. They also identified tumoral CRP expression as an independent factor for predicting poor clinical outcome. As to the first theory (i.e., elevated CRP levels cause cancer), however, Allin et al. [32] have only recently demonstrated its unlikelihood.

Our study has some significant limitations. First of all, its retrospective design precluded the systematic evaluation of important additional prognostic factors such as microscopic lymphovascular and perineural invasion, growth pattern, and anatomic site. The postoperative CRP value was not assessable for the majority of patients and therefore not included in this analysis. Moreover, we had only limited information about potential superinfections of the penile cancer which might have influenced the preoperative CRP value. The study also lacks a central pathologic review. In addition, the number of cases was relatively small (n = 79), and all potentially prognostic parameters included in the analysis were assessable in 54 patients, only; 48 of these patients had significant follow up to be included in the multivariable analysis. On the other hand, the study size still seems rather impressive, considering the rarity of penile cancer in Western countries, and exceeds that in many other penile cancer trials.

## Conclusion

In conclusion, we have shown that an elevated preoperative serum CRP level is significantly associated with reduced cancer-specific survival. However, since it is also significantly associated with other risk factors, particularly tumor stage, future studies with larger patient populations will have to clarify whether elevated CRP (1) can serve as an independent prognostic factor and (2) might improve the predictive accuracy of nomograms that will be developed in the future to optimally estimate and predict the prognosis of patients with penile cancer.

## Competing interests

We declare that we have no conflict of competing interest.

## Authors’ contributions

SS designed and planed the study, was part of the acquisition, analysis and interpretation of data and contributed to the darfting of the manuscript. AAG was part of the acquisition, analysis and interpretation of data and drafted the manuscript. JS, RL, TJS, MVC, GW and FJ were part of the data acquisition. MS and MAK were responsible for the supervision. AJS planed the study, was part of the acquisition, analysis and interpretation of data, performed the statistical analysis and drafted the manuscript. All authors read and approved the final manuscript.

## Pre-publication history

The pre-publication history for this paper can be accessed here:

http://www.biomedcentral.com/1471-2407/13/223/prepub
